# Material properties of the heel fat pad across strain rates

**DOI:** 10.1016/j.jmbbm.2016.09.003

**Published:** 2017-01

**Authors:** Grigoris Grigoriadis, Nicolas Newell, Diagarajen Carpanen, Alexandros Christou, Anthony M.J. Bull, Spyros D. Masouros

**Affiliations:** Department of Bioengineering, Imperial College London, London SW7 2AZ, UK

**Keywords:** Heel fat pad, Strain rate, Material properties, Hyperelasticity, Viscoelasticity, Foot and ankle

## Abstract

The complex structural and material behaviour of the human heel fat pad determines the transmission of plantar loading to the lower limb across a wide range of loading scenarios; from locomotion to injurious incidents. The aim of this study was to quantify the hyper-viscoelastic material properties of the human heel fat pad across strains and strain rates. An inverse finite element (FE) optimisation algorithm was developed and used, in conjunction with quasi-static and dynamic tests performed to five cadaveric heel specimens, to derive specimen-specific and mean hyper-viscoelastic material models able to predict accurately the response of the tissue at compressive loading of strain rates up to 150 s^−1^. The mean behaviour was expressed by the quasi-linear viscoelastic (QLV) material formulation, combining the Yeoh material model ($C_{10}=0.1\mathrm{MPa}$, $C_{30}=7\mathrm{MPa}$, K=2GPa) and Prony׳s terms ($A_{1}=0.06$, $A_{2}=0.77$, $A_{3}=0.02$ for $\tau_{1}=1\mathrm{ms}$, $\tau_{2}=10\mathrm{ms}$, $\tau_{3}=10s$). These new data help to understand better the functional anatomy and pathophysiology of the foot and ankle, develop biomimetic materials for tissue reconstruction, design of shoe, insole, and foot and ankle orthoses, and improve the predictive ability of computational models of the foot and ankle used to simulate daily activities or predict injuries at high rate injurious incidents such as road traffic accidents and underbody blast.

## 1 Introduction

The heel fat pad bears repeated loads during locomotion, spreads them over the calcaneus (the heel), and absorbs shocks (Buschmann et al., 1995, De Clercq et al., 1994, Jahss et al., 1992, Jørgensen and Bojsen-Møller, 1989, Ker et al., 1989). These functions depend on its material and structural behaviour, which is determined by its microstructure, geometry, and interface with surrounding tissues. With an average thickness of 18 mm from the calcaneus to the plantar skin (Bojsen-Møller and Jørgensen, 1991), the human heel fat pad contains a reticular arrangement of collagen/elastin fibrous walls that create compartments that surround and retain adipose tissue (Hsu et al., 2007, Jahss et al., 1992). Based on the size of these compartments, they can be categorised into two layers; superficial (attached to the plantar epidermis), and deep (attached to the calcaneus). The superficial layer contains micro-chambers while the deep layer consists of larger, macro-chambers (Blechschmidt, 1982).

The material and structural behaviour of the heel fat pad regulates the amount of load that is transmitted to the bones and joints of the lower limb across a range of loading scenarios; from low rate activities such as standing and running, to high rate incidents that can cause injury such as sport and vehicular accidents. Therefore, the characterisation of the tissue across a range of strain rates can be used for a variety of applications such as understanding the pathophysiology of related diseases (Kinoshita et al., 1996, Tong et al., 2003), shoe and insole design (Jørgensen and Bojsen-Møller, 1989), reconstruction of degenerated tissue (Wang et al., 1999), design of biomimetic materials for treatment of injuries and diseases of the plantar foot (Balkin and Kaplan, 1991, Mulder, 2012), development of accurate FE models (Fontanella et al., 2013), or prediction of and protection from injury in road traffic accidents and underbody blast (Dong et al., 2013, Shin et al., 2012).

The heel fat pad is inhomogeneous and anisotropic while it is reported to exhibit non-linear viscoelastic behaviour due to its biphasic nature (Rome, 1998). *In vivo* studies have used imaging (Gefen et al., 2001, Prichasuk, 1994), indentation (Erdemir et al., 2006, Rome et al., 2001), or both techniques (Tong et al., 2003) to quantify the material properties of the tissue. Indentation, however, cannot be utilised to obtain material properties as the captured behaviour depends on the diameter of the indenter and is localised (Spears and Miller-Young, 2006). Furthermore, the behaviour of the tissue cannot be investigated by *in vivo* experiments at high rates as they are likely to cause injury to the subject.

*In situ* (Aerts et al., 1996, Aerts et al., 1995, Bennett and Ker, 1990, Erdemir et al., 2009) and *in vitro* (Gabler et al., 2014, Ledoux et al., 2004, Miller-Young et al., 2002) testing of cadaveric heel fat pads permit the use of rigs and devices able to reach extreme and complex loading scenarios. *In situ* studies, however, report structural properties only in the form of force–displacement curves; these cannot generally express the behaviour of the material and have limited use as they strongly depend on the geometry of the tissue (Spears and Miller-Young, 2006), whilst *in vitro* testing requires disruption of the material continuity that may affect the material behaviour. The response of the tissue has been investigated for rates up to 60 s^−1^ (Gabler et al., 2014), however, there exist situations, for example in under-body blast, that the tissue can be loaded at rates quicker than this (Bir et al., 2008).

In order to overcome the complications of *in vivo*, *in situ* and in *vitro* methods in obtaining the material properties of the heel fat pad, computational studies and inverse FE modelling can be used. Inverse FE modelling is an optimisation procedure attempting to minimise the difference between captured data from experiments and the numerical results from FE simulations replicating the experimental protocol. The combination of experimental and computational work permits thorough investigation of the response of the tissue without the need to isolate small samples and disrupt its material continuity. Although this has been attempted previously for the heel fat pad, these studies have adopted simple, 2D FE models (Erdemir et al., 2006, Spears and Miller-Young, 2006), or reported a complicated material behaviour expressed by a formulation that is not supported by commercial FE software packages and predicts tissue behaviour only at low loading rates (Natali et al., 2012).

The aim of this study was to quantify the material properties of the human heel fat pad across a range of strains and strain rates using an inverse FE method in conjunction with experiments whereby the fat pad structure and its interface with surrounding tissues was not disrupted.

## 2 Methods

### 2.1 Sample preparation

Five male cadaveric lower extremities (mean age 47 years, range 40–57 years), with no known pathology that could affect the properties of the fat pad, were obtained from a licensed human tissue facility. Ethical approval was obtained from the Tissue Management Committee of the Imperial College Tissue Bank ethics committee (Ethical approval number: 12-WA-0196).

The specimens were stored fresh frozen at −20 °C and thawed prior to dissection and testing. Each specimen was Computed Tomography (CT) scanned (Siemens Somatom Definition AS 64, Erlangen, Germany) with a slice thickness of 1 mm and an in plane pixel size of 0.4×0.4 mm^2^ (voxel size of 0.16 mm^3^) in order to check for any pre-existing orthopaedic pathology and to obtain geometric data for the FE models.

Feet were dissected from the lower extremities by sectioning along a transverse plane proximal to the distal end of the tibial diaphysis, such that both distal tibia and fibula were preserved. To isolate the calcaneus with the fat pad attached and to prepare the sample for potting, a custom-built rig was used to ensure that the sample was positioned in a typical standing, or seated posture. This involved resting the sole of the foot flat against the bottom of the rig and positioning the exposed distal tibia perpendicular to the bottom of the rig. At this stage, soft tissues covering the medial and lateral sides of the calcaneus were removed to permit bolts to be squeezed against the bone to hold the specimen in place. The distal tibia, the fibula, the forefoot anterior to the calcaneus, the talus, and the cartilage of the posterior and anteromedial facets were removed to reveal the proximal surface of the calcaneus.

**Fig. 1:**
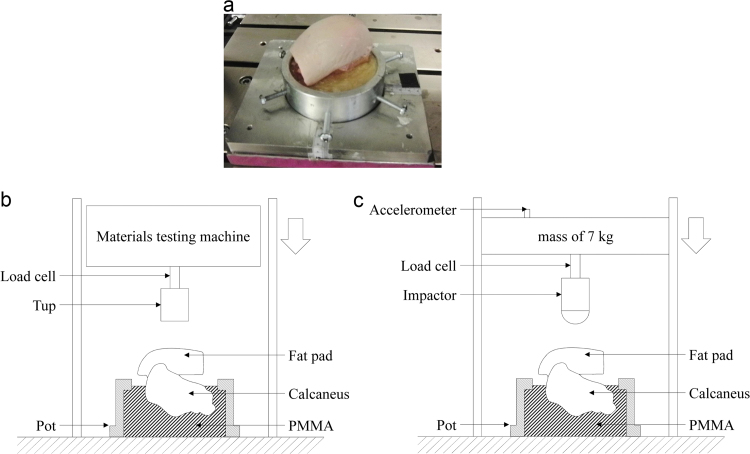
(a) Photograph of the prepared sample, potted in PMMA and held in the potting ring. (b) and (c) show schematics of the apparatus used for conducting quasi-static and high rate compressive testing, respectively.

Each sample, still secured on the dissection rig, was turned upside down and fixed with clamps such that approximately half of the calcaneal body was below the edge of a 45 mm deep cylindrical pot. The sample was fixed into position within the pot using polymethyl methacrylate (PMMA) bone cement (Fig. 1a). Four uniaxial strain gauges (model C2A-06-125LW-120, Vishay PG, Bradford, UK) were attached to the calcaneal body using cyanoacrylate in order to monitor the deformation of the bone and detect fracture. Two were positioned on the medial and two on the lateral side, and on each side one gauge was aligned vertically and one horizontally. Throughout preparation and testing, the samples were regularly sprayed with phosphate-buffered saline (PBS) to keep them hydrated.

### 2.2 Compressive testing

Each sample was subjected to both quasi-static and dynamic testing. Although similar methods were used for both types of testing, the testing rig and loading protocols differed and are therefore described separately in the following sections.

#### 2.2.1 Quasi-static tests

Quasi-static compression tests were carried out using a screw-driven uniaxial materials testing machine (5866 series, Instron, High Wycombe, UK). Each sample was centred beneath a cylindrical, flat tup, 50 mm in diameter that was connected to a 10 kN load cell (resolution ±1 N) that was incorporated in the machine (Fig. 1b). Load, displacement and strain were recorded at a frequency of 1 kHz using a PXIe data acquisition system (model 1082, National Instruments, Austin, TX, USA) and a custom-written LabVIEW code (v2012, National Instruments, Austin, TX, USA).

For the quasi-static compression tests, three preconditioning compressive cycles were performed up to 5 N before the fat pad tissue was compressed to 50% strain at a speed of 0.01 mm/s. This protocol was repeated twice for each sample; between tests, samples were allowed to rest for 15 min. Preliminary investigations demonstrated that 15 min resting time and three preconditioning cycles were sufficient to ensure that the behaviour of the fat pad was consistent; the force–displacement data from the repeated tests after the second preconditioning cycle were similar for all samples (relative error less than 2% and *R*^2^>0.99). The displacement required to achieve the target strain was calculated using the undeformed thickness of the tissue measured from the CT scans.

#### 2.2.2 Dynamic tests

Dynamic tests were performed using a drop rig (Dynatup 9250 HV, Instron, High Wycombe, UK) on the same day after the quasi-static tests. The drop rig incorporates a load cell (40 kN capacity, resolution ±5 N) above a cylindrical, curved 50 mm diameter tup (Fig. 1c). A curved tup was used for the dynamic tests to avoid disruption of the tissue that may have been caused by sharp edges at high rates. An accelerometer (model 352C04, PCB Piezotronics Ltd, Hitchin, UK) was secured to the top of the 7 kg falling mass and was used to calculate the velocity and position of the impactor during testing. High speed video (Phantom V12.1, 33000 fps, Vision Research, Bedford, UK) was captured to confirm the time of initial contact and help determine time of failure.

Tests were performed at increasing drop heights of 2, 4, 8, 16, 32 and 64 cm, corresponding to target velocities at impact of 0.6, 0.85, 1.2, 1.7, 2.4 and 3.4 m/s. To confirm that the sample was not damaged during the tests, after each increase in drop height a repeat of the initial 2 cm drop test was performed and the peak force, time to peak force and slope of the force–time curve were compared. If the difference in any of these parameters between initial and repeated 2 cm drop was greater than 10%, the sample was deemed to have failed and testing was stopped.

All data were recorded using the same data acquisition system that was used for the quasi-static tests, however, at a frequency of 25 kHz. A low-pass Butterworth filter was used to filter the force measurements. The cut-off frequency (1 kHz) was selected based on the frequency analysis of the signal.

### 2.3 FE modelling

#### 2.3.1 Geometry extraction and meshing

**Fig. 2:**
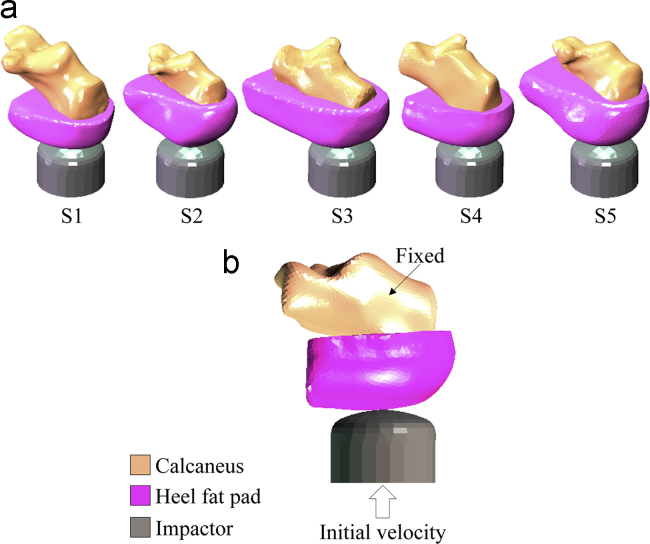
(a) The specimen-specific models of all samples. (b) The boundary conditions of the FE simulation of the dynamic tests for one of the samples.

Specimen-specific finite element models were developed to simulate the quasi-static and dynamic tests (Fig. 2a). The geometries of calcaneus and heel fat pad of each sample were extracted from the CT scans using Mimics (v15.0, Materialise HQ, Leuven, Belgium).

The extracted geometries were imported as stereolithography (.stl) files in Geomagic Studio (v2013, Geomagic Inc., Morrisville, NC, USA) to form solid objects that were then processed in Autodesk Inventor (v2013, Autodesk Inc., San Rafel, CA, USA) to perform Boolean operations and achieve a good match between contacting surfaces. The heel fat pad was meshed with Herrmann tetrahedral finite elements (Herrmann, 1965) using HyperMesh (v13.0.110, Altair, Troy, MI, USA) with an average element side length of 1.5 mm, which was found to be sufficient for creating a converged mesh. The cortical calcaneus was modelled as a rigid surface and both the bone and the fat pad were imported into the nonlinear FE software package MSC.Marc (v2014, MSC.Software, Santa Ana, CA, USA) to setup and run the simulations. Both cortical and trabecular structures as well as the surrounding bone cement were initially meshed and modelled with tetrahedral finite elements for every sample. A sensitivity analysis was performed which showed that their behaviour did not affect the force–time response of the model and so they were replaced by a rigid surface to reduce the computational cost.

#### 2.3.2 Boundary conditions

The cortical calcaneal bone was fixed while the tup, modelled as a rigid body, was restricted to move in the direction of the impact only. For the quasi-static compression simulations the tup was displacement driven to compress the samples up to the target displacement. The input for the dynamic simulations was the initial velocity of the impactor prior to coming into contact with the sample (Fig. 2b); for each simulation the initial velocity was set to be equal to the velocity at impact of the respective dynamic test. The mass of the impactor was 7 kg as in the experimental apparatus.

Contact between fat pad and calcaneus was set to ‘glued’ thereby slipping was not allowed. ‘Touching’ contact was implemented between tup and fat pad; the fat pad was allowed to slide on the rigid surface with a low coefficient of friction (0.01). A sensitivity analysis showed that coefficient of friction values between 0.005 and 1.5 – a physiological range of the coefficient of friction between palmar skin and several types of metal, reported by O’Meara and Smith (2001) – did not alter the force experienced by the tup (less than 1% relative error).

#### 2.3.3 Inverse FE algorithm

An inverse FE algorithm was used to determine the strain rate dependent material properties. The algorithm is based on the derivative free Nelder-Mead or downhill simplex method for function minimisation (Nelder and Mead, 1965) and was developed using a combination of programming languages (Fortran, Matlab, Python) and MSC.Marc. This algorithm can be used to find the local minimum or maximum of an objective function specified by the user. The main output of numerical and experimental tests was the compressive force measured over time at the plantar fat pad by the load cell. Therefore, the objective function was formed to calculate and minimise the difference between the experimental ($x_{\exp}$) and numerical ($x_{\mathrm{num}}$) force measurements (Eq. 1). A factor (δ) (Eq. 2) was also included in the mathematical formula to ensure that force measurements that were less than 1 N did not contribute as significantly to the objective function as those above 1 N.

(1) $$O.F.=\sum_{i=1}^{n}\left(\delta ∙{\left(\frac{x_{\exp,i}-x_{\mathrm{num},i}}{x_{\exp,i}}\right)}^{2}+\left(1-\delta \right)∙{\left(x_{\exp,i}-x_{\mathrm{num},i}\right)}^{2}\right)$$

(2) $$\delta =\{\begin{array}{c} 0,x_{\exp,i}<1\\ 1,x_{\exp,i}\geq 1 \end{array}$$

The formulation that was selected to represent the hyperelasticity of the tissue was a subcategory of the generalised Mooney–Rivlin material model, the Yeoh material formulation described in Eq. (3) (Bonet and Wood, 2008).

(3) $$W=\sum_{i=1}^{3}C_{i0}{\left(I_{1}-3\right)}^{i}+\frac{9}{2}K{(J^{1/3}-1)}^{2}$$

The material constants $C_{10}$, $C_{20}$, $C_{30}$ of the tissue were the optimising parameters of the procedure while $I_{1}$ and J represent the first Cauchy–Green strain invariant and the volumetric deformation, respectively. The value assigned to the bulk modulus, K (2 GPa), was defined from a preliminary sensitivity analysis in order to ensure incompressible behaviour of the material and it was within the range of values reported in the literature for the same (Gabler et al., 2014), or other incompressible biological tissues (Etoh et al., 1994, Glozman and Azhari, 2010). The optimisation algorithm was considered converged when the change in objective function and material constants in consecutive iterations was less than 0.001% and 0.0001 MPa, respectively.

In order to investigate the strain rate dependency of the above formulation, only the initial part of the force–time history curves from each dynamic experiment were used in the objective function (up to until the velocity of the impactor had dropped by 10%); during this time the strain rate of the sample can be assumed to be constant. By using this method, each test corresponded to a different strain rate and each run of the optimisation algorithm gave a set of specimen-specific material properties for each sample.

By the end of the optimisation procedure, a set of material constants $C_{10}$, $C_{20}$, $C_{30}$ had been derived for each strain rate and every sample. In order to allow information from each of the material models to be used in simulations of varying strain rate, a custom-written user subroutine was implemented to each of the specimen-specific FE models. The user subroutine ensured that the appropriate material properties were assigned to the fat pad for each increment of the simulation depending on the strain rate experienced by the tissue at the previous step. This was achieved by linearly interpolating across the material constants derived for constant strain rates. Using this method, five specimen-specific material models were implemented into the FE models.

The properties were averaged across all samples in order to provide a material formulation described by strain rate dependent relationships of the material constants ($C_{10}\left(\dot{\varepsilon }\right),C_{20}\left(\dot{\varepsilon }\right),C_{30}\left(\dot{\varepsilon }\right)$). These cannot be implemented without the use of custom-written scripts in models simulating load cases of varying strain rate. To derive a continuous material formulation supported by most FE software packages, the QLV material formulation was used (Fung, 1981). Five terms of a Prony series (relaxation constants $A_{k}$, time constants $\tau_{k}$) were included in the Yeoh model and fitted to the average hyperelastic and strain rate dependent material formulation (Eq. 4).

(4) $$W=\int_{0}^{t}\left[\sum_{i=1}^{3}C_{i0}\left(1-\sum_{k=1}^{5}A_{k}\left[1-e^{\left(-\frac{t-\tau }{\tau_{k}}\right)}\right]\right)\frac{d}{d\tau }{\left(I_{1}-3\right)}^{i}\right]d\tau$$

The specimen-specific and the average QLV material formulations were finally implemented in MSC. Marc to simulate each dynamic test for its whole duration and compare the result against the experimental data. During the dynamic tests the tissue experienced a range of strain rates. A fit between numerical and experimental results on each sample for the highest, non-catastrophic dynamic test was used to test the derived formulation for validity since the largest range of strain rates was expected in this test.

## 3 Results

### 3.1 Quasi-static tests

**Fig. 3:**
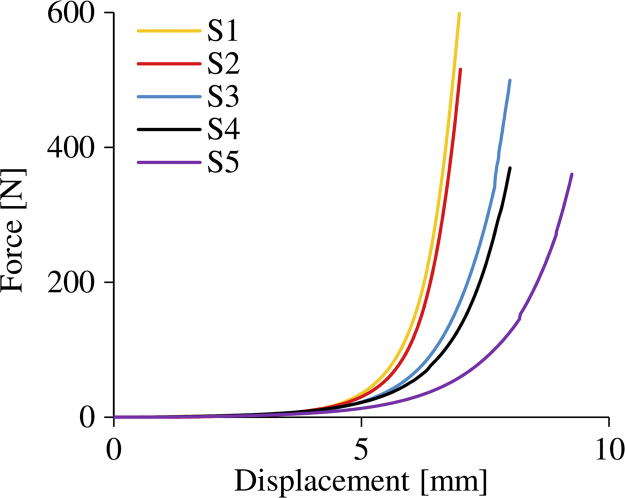
The quasi-static compressive force–displacement curves for all 5 samples.

The compressive force–displacement curves for all samples are shown in Fig. 3. All samples exhibited hyperelastic behaviour under quasi-static compression up to 50% strain with maximum forces ranging from 369 to 616 N.

### 3.2 Dynamic tests

**Fig. 4:**
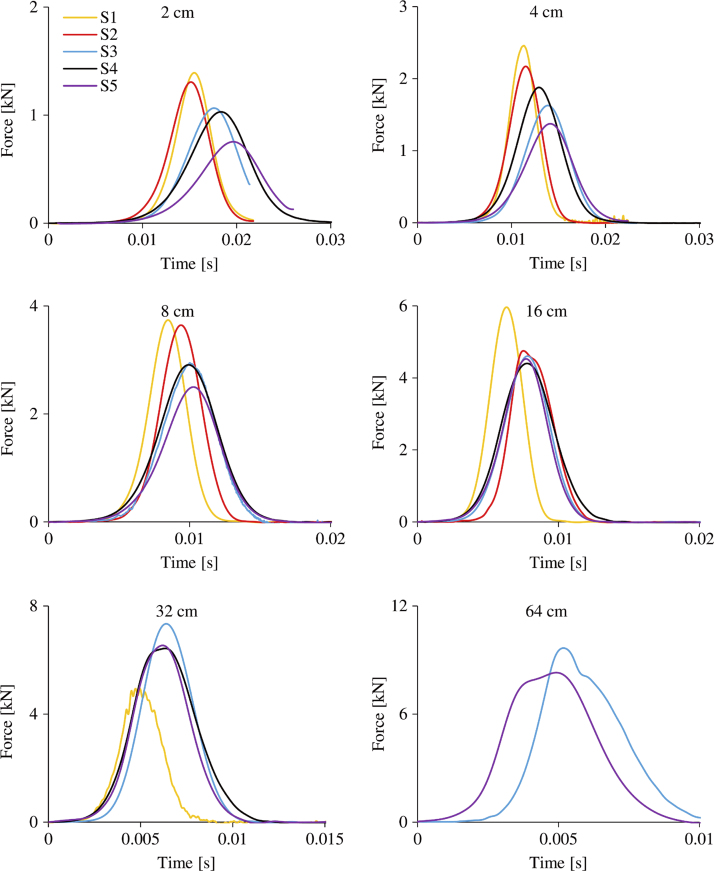
The force–time history curves from dynamic tests from all drop heights for all 5 samples.

The force–time history curves for all samples at all drop heights are presented in Fig. 4. Two samples failed at the last drop from the height of 64 cm, two at the 32 cm and one at the 16 cm drop. The mean maximum compressive force that was reached prior to failure was 6.52 (SD 1.96) kN.

### 3.3 Inverse FE optimisation

**Fig. 5:**
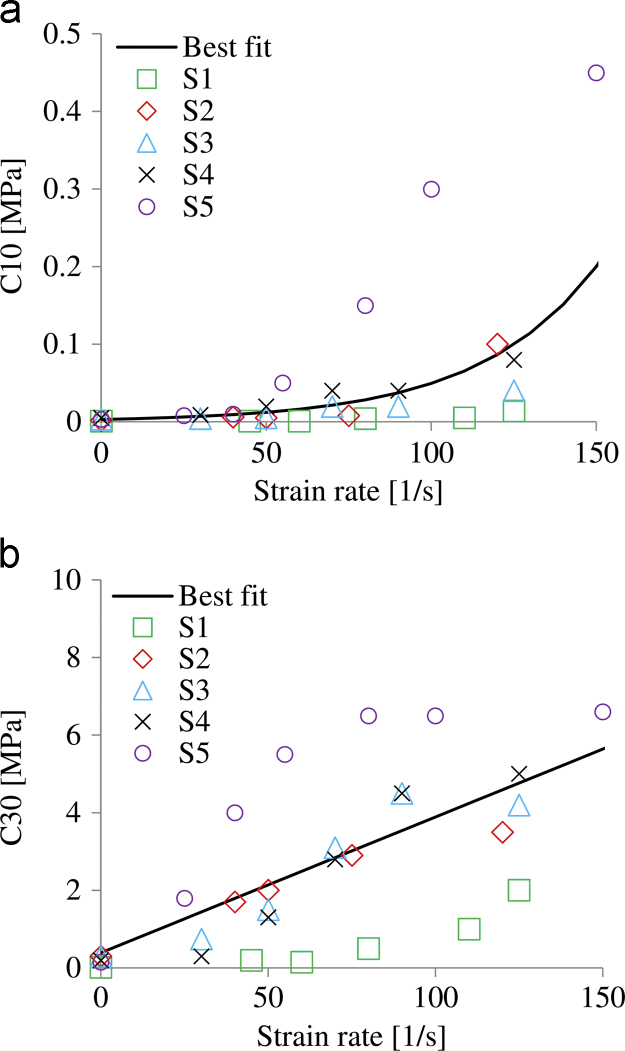
Derived material constants (a) *C*_10_ and (b) *C*_30_ and the respective best fitted curves for all samples and rates.

The derived values for the material parameters $C_{10}$ and $C_{30}$ are shown in Fig. 5. The derived values for the material parameter $C_{20}$ were consistently less than 0.0001 MPa and therefore the term was set to zero.

**Fig. 6:**
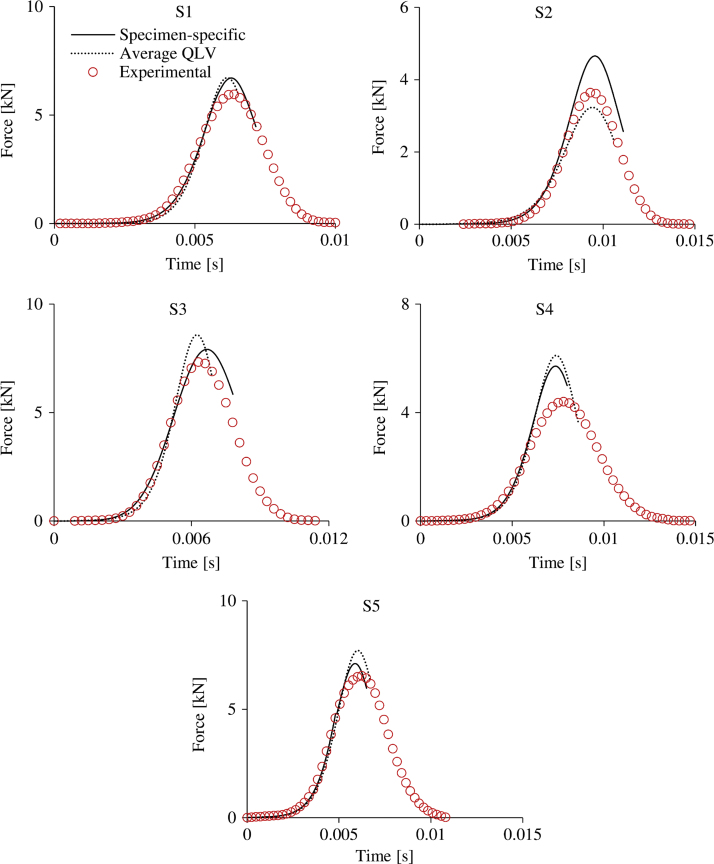
Comparison between the experimental and computationally predicted (using both specimen-specific $C_{10}\left(\dot{\varepsilon }\right)$ and $C_{30}\left(\dot{\varepsilon }\right)$ values and the QLV model) force–time curves from the fastest non-catastrophic test of each sample.

The $C_{10}\left(\dot{\varepsilon }\right)$ and $C_{30}\left(\dot{\varepsilon }\right)$ relationships that were best fitted to the derived material parameters for all samples are described by Eqs. (5), (6) and shown in Fig. 5. The material properties of the QLV model that were best fitted (*R*^2^=0.84) to the average strain rate dependent $C_{10}\left(\dot{\varepsilon }\right)$ and $C_{30}\left(\dot{\varepsilon }\right)$ relationships are shown in Table 1. From the five Prony׳s terms, through the fitting procedure, two terms got values lower than 0.00001 and were neglected. The ability of the $C_{10}\left(\dot{\varepsilon }\right)$ and $C_{30}\left(\dot{\varepsilon }\right)$ relationships and the QLV material model to predict accurately the experimental result is compared in Fig. 6.

(5) $$C_{10}=0.003e^{0.028\dot{\varepsilon }}(R^{2}=0.49)$$

(6) $$C_{30}=0.035\dot{\varepsilon }+0.39,(R^{2}=0.5)$$

## 4 Discussion

This study has characterised the material properties of the heel fat pad across the largest range of strain rates to date and is the first to attempt to identify properties for rates higher than 60 s^−1^. An inverse FE method was used such that the material continuity of the tissue was not disturbed. This method combines the benefits of *in situ* and *in vitro* testing as stress–strain curves can be obtained through testing the whole area of interest and not isolated components. Although previous studies also utilised inverse FE methods for the same purposes (Erdemir et al., 2006, Natali et al., 2012, Spears and Miller-Young, 2006), this is the first study where the inverse algorithm was based on specimen-specific FE models.

All samples exhibited hyperelastic and strain rate dependent behaviour; the tissue was found to exhibit a stiffer response with both higher strain and higher strain rate. This is in agreement with the majority of previous experimental studies (Bennett and Ker, 1990, Erdemir et al., 2006, Gabler et al., 2014, Ledoux and Blevins, 2007, Miller-Young et al., 2002). An average strain-rate dependent formulation and a QLV model were derived to capture that behaviour and can be implemented readily in FE models used to simulate various load cases; from daily activities to high rate road traffic accidents or under-body blast. No limit above which the behaviour becomes independent of the strain rate was identified. However, as shown in Fig. 5, above strain rates of 70 s^−1^ smaller changes in material constants are seen in all samples apart from S1 and S5 for $C_{30}$ and $C_{10}$, respectively. Therefore, it is likely that at a higher rate a limit would have been reached.

The specimen-specific data shown in Fig. 5 are associated with high variability. This is also highlighted by the low coefficient of determination ($R^{2}\leq 0.5)$ of the best fitted strain-rate dependent $C_{10}\left(\dot{\varepsilon }\right)$ and $C_{30}\left(\dot{\varepsilon }\right)$ relationships (Eqs. (5), (6)). This finding suggests that in future applications specimen-specific data should be preferred when available. Conducting experiments on a greater amount of samples is highly recommended in order to investigate further whether the material behaviour of the heel fat pad can be represented appropriately by an average material formulation. Despite the high variability, the derived average QLV material model provided results as accurate as the specimen-specific formulations for a high rate simulation (Fig. 6).

**Fig. 7:**
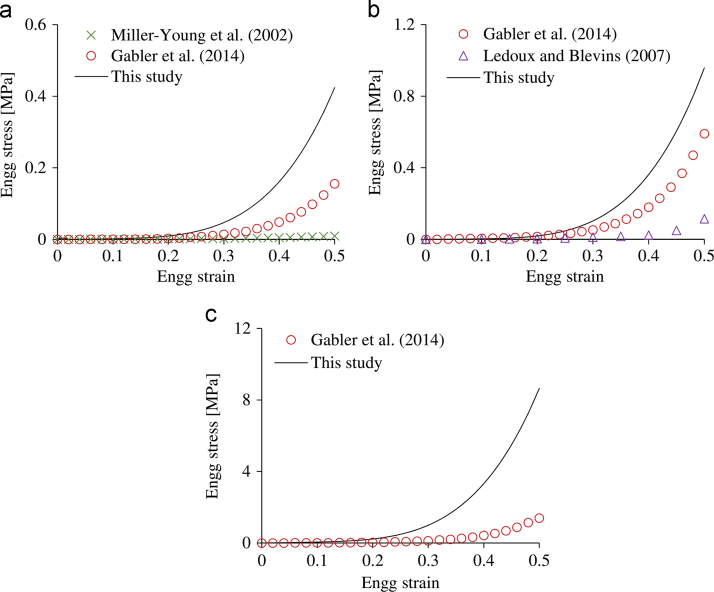
(a) Comparison between the average compressive engineering (Engg) stress–strain curve of the human fat pad derived in this study and in previous attempts for (a) quasi-static, (b) 10 s^−1^ and (c) 100 s^−1^ strain rates.

The results obtained in this study are compared to previous studies in Fig. 7. Stress–strain curves, which are independent of the material geometry and were reported mostly by *in vitro* studies, were selected for comparing as the force–displacement response of the heel fat pad depends on the thickness and the area over which the force is applied. The average material formulation of the heel fat pad for quasi-static (Fig. 7a) and dynamic (10 s^−1^ and 100 s^−1^) (Fig. 7b&c) strain rates derived in this study describes consistently stiffer behaviour than previously reported by *in vitro* studies (Gabler et al., 2014, Ledoux and Blevins, 2007, Miller-Young et al., 2002). In the case of the Miller-Young et al. (2002) study this could be due to the diameter (8 mm) of the samples tested being slightly smaller than the average thickness of the macro-chambers of the unloaded tissue (10 mm; Hsu et al., 2007). The average behaviour obtained in this study for a strain rate of 10 s^−1^ is close to the behaviour suggested by Gabler et al. (2014) (Fig. 7b). However, there is a marked difference between the material behaviours at 100 s^−1^. This may be due to the fact that the tests conducted by Gabler et al. (2014) went up to a maximum strain rate of 60 s^−1^ and the material response for higher rates was extrapolated.

A potential limitation of cadaveric studies is that the behaviour of living tissue might differ from cadaveric tissue. One of the major differences between living and cadaveric tissue is the blood propulsion; this has been shown not to affect the behaviour of the heel fat pad for compression rates higher than 0.4 m/s while at lower rates it does not affect the stiffness of the tissue more than 3% (Weijers et al., 2005). Bennett and Ker (1990) also showed that freezing of the tissue does not affect its behaviour as results from samples tested immediately post-amputation did not differ from those after freezing and thawing the same samples. Since the loading rates of this study are impossible to reach with an *in vivo* protocol, testing of cadaveric tissue is the most appropriate methodology. Compared to results from an *in vivo* quasi-static experiment utilising imaging techniques (Gefen et al., 2001), the material behaviour reported in this study is stiffer by one order of magnitude. Based on the findings mentioned above (Weijers et al., 2005) this is more likely to be due to the different experimental settings rather than the fact that cadaveric samples were used in this study. A possible reason for this discrepancy could be the fact that despite using an accurate imaging technique, the strain calculations assumed a uniform and uniaxial strain distribution. The use of FE modelling overcomes this limitation as the deformation of the tissue can be realistically represented; non uniform and in various directions. The possibility that the use of cadaveric tissue determined the outcome of this study cannot be definitely rejected, especially since contradicting results from testing living and cadaveric heel fat pads have been also reported in previous studies (Kinoshita et al., 1996, Bennett and Ker, 1990). This discrepancy has been considered as a paradox by Aerts et al. (1995) and was addressed to the difficulty of isolating the response of the heel fat pad from that of the whole human body in an *in vivo* setting.

The temperature of the sample during testing was equal to the room temperature (20–25 °C). Bennett and Ker (1990) reported that between this temperature and the physiological body temperature (37 °C) the dissipation ability of the tissue drops by less than 3%. Therefore, the effect of this factor on the material properties is minimal.

The suggested formulations describe the combined response of the soft tissue underneath the calcaneus (micro and macro-chambers, adipose tissue, skin). This is due to the fact that the plantar soft tissues of the heel, attached to the calcaneus, were not disrupted prior to testing and were segmented as a single, homogenous structure from the CT scans. This is not a limitation for the suggested applications where the structural response of the soft tissues of the heel region are of interest.

The average and specimen-specific material models predict well the slope of the force–time history curve for the drops on each sample but not the peak force and the unloading curve of the graph. When the strain rate of the simulated test becomes less than 10 s^−1^ the FE models overestimate the experimental response. The physiological explanation of this mismatch is associated with the balance between the response of the fibrous tissue and the fat globules. When compressed slowly, the fat globules expand circumferentially and stretch the chambers that mainly restrict this motion. The response is expected to be different when the tissue is compressed slowly at the end of a dynamic test after the tissue had been deformed rapidly and mainly the fat globules have been restricting the deformation up to this point. Numerically, this difference can be explained by the lack of energy dissipation terms for both material formulations. Although the strain rate dependence is taken into account, when the specimen-specific formulations are implemented, the tissue unloads like a spring and returns all the stored energy. Although the implemented QLV model includes energy dissipation terms (Prony series), it was best fitted to the specimen-specific, strain-rate dependent formulations and therefore the accuracy did not improve. This limitation can be tackled by adopting a different optimisation strategy where the QLV material parameters are optimised directly for all dynamic tests of each sample simultaneously.

The results from this study are important for understanding heel biomechanics at various conditions, from daily activities such as walking and running to high rate loading scenarios involving injury. The derived properties can be implemented readily in FE models of the foot and ankle used for a wide range of applications; from shoe and insole design to injury prediction and design of protection. The accurate description of the material behaviour of the fat pad tissue also permits better selection of materials that can be used for reconstruction. Finally, the novel inverse FE method developed can be used to characterise the material behaviour of other complex biological tissues, such as brain tissue or the intervertebral disc, across strain rates and for various types of loading.

## Figures and Tables

**Table 1:** Values of the average QLV material formulation of the heel fat pad.

$C_{10}$ [MPa]	$C_{30}$ [MPa]	K [GPa]	$A_{1}$ for $\tau_{1}=1\mathrm{ms}$	$A_{2}$ for $\tau_{2}=10\mathrm{ms}$	$A_{3}$ for $\tau_{3}=0.1s$	$A_{4}$ for $\tau_{4}=1s$	$A_{5}$ for $\tau_{5}=10s$
0.1	7	2	0.06	0.77	0	0	0.02
